# Exposure to traffic-related air pollution and changes in exhaled nitric oxide and DNA methylation in arginase and nitric oxide synthase in children with asthma

**DOI:** 10.1186/s12940-020-00678-8

**Published:** 2021-02-11

**Authors:** N. Ji, M. Fang, A. Baptista, C. Cepeda, M. Greenberg, I. Colon Mincey, P. Ohman-Strickland, F. Haynes, N. Fiedler, H. M. Kipen, R. J. Laumbach

**Affiliations:** 1 Rutgers, The State University of New Jersey, 170 Frelinghuysen Rd, Room 204, Piscataway, NJ 08854 USA; 2 The New School, New York, NY USA; 3 Ironbound Community Corporation, Newark, NJ USA

**Keywords:** Traffic-related air pollution, DNA methylation, Asthma, Exhaled nitric oxide

## Abstract

**Background:**

Traffic-related air pollution (TRAP) has been associated with increased risk of airway inflammation in children with asthma. While epigenetic changes could potentially modulate TRAP-induced inflammatory responses, few studies have assessed the temporal pattern of exposure to TRAP, epigenetic changes and inflammation in children with asthma. Our goal was to test the time-lag patterns of personal exposure to TRAP, airway inflammation (measured as fractional exhaled nitric oxide, FeNO), and DNA methylation in the promoter regions of genes involved in nitric oxide synthesis among children with asthma.

**Methods:**

We measured personal exposure to black carbon (BC) and FeNO for up to 30 days in a panel of children with asthma. We collected 90 buccal cell samples for DNA methylation analysis from 18 children (5 per child). Methylation in promoter regions of nitric oxide synthase (*NOS1, NOS2A, NOS3*) and arginase (*ARG1, ARG2*) was assessed by bisulfite pyrosequencing. Linear-mixed effect models were used to test the associations of BC at different lag periods, percent DNA methylation at each site and FeNO level.

**Results:**

Exposure to BC was positively associated with FeNO, and negatively associated with DNA methylation in *NOS3*. We found strongest association between FeNO and BC at lag 0–6 h while strongest associations between methylation at positions 1 and 2 in *NOS3* and BC were at lag 13–24 h and lag 0–24 h, respectively. The strengths of associations were attenuated at longer lag periods. No significant associations between exposure to TRAP and methylation levels in other *NOS* and *ARG* isoforms were observed.

**Conclusions:**

Exposure to TRAP was associated with higher levels of FeNO and lower levels of DNA methylation in the promoter regions of the *NOS3* gene, indicating that DNA methylation of the *NOS3* gene could be an important epigenetic mechanism in physiological responses to TRAP in children with asthma.

**Supplementary Information:**

The online version contains supplementary material available at 10.1186/s12940-020-00678-8.

## Background

Exposure to traffic-related air pollution (TRAP) has been linked to a number of adverse health outcomes, and the Health Effect Institute (HEI) concluded that exposure to TRAP is causally associated with asthma exacerbation among children [1]. Here, we define TRAP as the air pollutants emitted from motor vehicle engines. Major constituents of TRAP include carbon monoxide (CO), oxides of nitrogen (NOx), volatile organic compounds (VOCs), and particulate matter (PM). Black carbon (BC) is a relatively specific marker for diesel engine-derived TRAP that is ubiquitous in urban communities and typically more intense near major roadways, seaports, and other centers of commercial activity. Airway inflammation is a key step in a hypothesized mode of action by which exposure to TRAP is thought to exacerbate asthma. Higher level of fraction of exhaled nitric oxide (FeNO), a marker of airway inflammation, was observed after short-term exposure to TRAP [2–4]. Elevated level of FeNO has, in turn, been associated with airway hyperresponsiveness and exacerbation of asthma [5]. These findings suggest that nitric oxide (NO) production in the respiratory tract plays an important role in asthmatic airway’s response to TRAP.

There is increasing evidence that TRAP-induced changes in FeNO may at least partly result from epigenetic modifications that alter the expression of genes involved in the synthesis of NO. NO can be synthesized from L-arginine by three NO synthase (*NOS*) isoforms: (a) neuronal *NOS* (*nNOS* encoded by *NOS1*); (b) inducible *NOS* (*iNOS* encoded by *NOS2A*); and (c) endothelial *NOS* (*eNOS* encoded by *NOS3*) [6]. In addition, there are two isoforms of Arginase (*ARG*) (encoded by *ARG1* and *ARG2*), which compete with *NOS* for the L-arginine substrate [7]. Therefore, epigenetic modifications that alter the expression of *NOS* and *ARG* may modulate production of NO in the respiratory tract and hence FeNO.

Exposure to air pollution has been associated with changes in DNA methylation in the promoter regions of *NOS* or *ARG* suggesting a mode of action by which air pollution may alter NO production and FeNO. However, the current epidemiological evidence regarding exposure to TRAP and changes in FeNO and DNA methylation has three main limitations when establishing a chain of causation in acute exacerbation of asthma. First, the temporal sequence of TRAP, *NOS* and *ARG* methylation and FeNO is necessary when inferring causality. However, only five observational studies have examined the temporality [8–12]. Second, all but one of these studies relied on fixed-site measurements in assessing individual-level exposure, which may have biased associations between TRAP and respiratory outcomes compared to personal exposure measurements [12]. Third, none of the five studies focused on children with asthma, for whom respiratory inflammation plays a critical role in the exacerbation of this disease. One cohort study assessed the impacts of DNA methylation on FeNO in children [13]. They found that the strength of association between DNA methylation in ARG and FeNO was stronger in children with asthma compared to those without asthma.

We performed a longitudinal panel study, aimed to assess the effects of exposure to TRAP on FeNO and DNA methylation in promoter regions of *NOS* and *ARG* genes. We sampled buccal cells from children with asthma living in an urban, seaport-adjacent community in New Jersey. Using real-time measurement of personal exposure to black carbon as an indicator for TRAP exposure, we investigated the temporal patterns of DNA methylation and FeNO responses. We hypothesized that exposure to TRAP would be positively associated with the FeNO levels and methylation of *ARG* genes, and negatively associated with methylation of *NOS* genes. We further hypothesized that the associations between TRAP and methylation would occur within a shorter lag period than the associations between TRAP and FeNO.

## Methods

### Study population

This study was nested in a panel study with repeated measurements on TRAP and FeNO. From 2011 to 2016, we repeatedly sampled 36 children from non-smoking households living in Newark or Elizabeth NJ with mild intermittent to moderate asthma by The National Heart, Lung, and Blood Institute (NHLBI) criteria [14]*.* All of the participants were taking an inhaled bronchodilator and/or inhaled corticosteroid, which was uninterrupted during the study*.* Each subject was followed for up to 30 days. Power calculations to examine the within-subject association between either exposure and methylation, or methylation and FeNO, were conducted via simulation. Simulations assumed 18 subjects with an average of 5 repeated measures per subject, using a sum across subjects of the fisher-transformed correlations within subject, which follows a normal distribution. Assuming we conducted a two-sided hypothesis test that the correlation was equal to zero, we would have 75.9% power to detect a correlation of 0.35. For within-subject correlations of 0.40 and 0.45, we would have powers of 85.7 and 92.1%, respectively. Therefore, a subset of 18 children (5 buccal samples per child) was randomly selected for the DNA methylation study. The inclusion criteria are: subjects with at least five buccal cell samples; with FeNO measurements, and with less than 20% missing of 24-h BC data. The Institutional Review Board of Rutgers University approved the epigenetic study protocol. Parents or legal guardians provided informed consent for all subjects.

### FeNO measurements

FeNO was measured using the NIOX MINO (Aerocrine, New Providence, NJ) following the manufacturer’s instructions and the standards of the American Thoracic Society and the European Respiratory Society. Concentrations of NO were measured during 10-s exhalations of breath at an exhalation pressure of 10–20 cmH_2_O to maintain a flow rate of 50 ± 5 ml/second. To control for the possible circadian effects, FeNO measurements were conducted at the same time (around 4:00 p.m.) for all subjects on the same day when collecting buccal cell samples.

### Buccal cell samples

Buccal cell samples were collected at the community field site on various weekdays to better capture the variation in DNA methylation on different day-of-week. Each child was provided with two toothbrushes. They were instructed to remove all food from their mouths and used the first toothbrush to brush their teeth before buccal cell collection. After that, children were instructed to gently brushed buccal mucosa 10 strokes with the second toothbrush and then to rinse the mouth with water for 30 s. Then the subjects were told to spit the content in a tube and put the second brush in the expelled water. Isopropyl alcohol (70%) was added to the tube after sampling. In the lab, buccal cell suspensions were centrifuged at 2500 rpm (1000 g) for 15 min. The pellets were stored frozen at − 80 °C until used for DNA extraction.

### DNA methylation

We studied CpG loci located in promoter regions of *NOS1* (6 CpG sites), *NOS2A* (16 CpG sites), *NOS3* (3 CpG sites), *ARG1* (8 CpG sites) and *ARG2* (13 CpG sites) genes. The rationale of selecting the location of the gene promoter, amplified regions, and CpG sites were supported by another study by Breton et al. [13]. Lab technicians who performed DNA methylation analysis were blinded to subject information. Genomic DNA was extracted from the cell pellets with Quick-DNA 96 Kit (Zymo Res.) and 1 μg DNA subjected to bisulfite conversion with EZ-96 DNA Methylation Kit (Deep-Well) (Zymo Res.) for downstream analyses, including polymerase chain reaction (PCR) and Pyrosequencing. Samples were then amplified by PCR and analyzed using a PSQHS96 Pyrosequencing System (EpigenDx, Hopkinton, MA). Methylation status at each CpG site was measured by calculating the ratio of C (methylated cytosine) relative to T (unmethylated cytosine) [15]. For each CpG site tested, the percentage of 5-methylcytosine (%5mC) was calculated and presented as the degree of methylation. For all assays, methylated and unmethylated DNA standards were used as positive and negative control, respectively to validate the method of bisulfite conversion and PCR amplification.

### Air pollutants

Personal real-time BC data was recorded by micro-aethalometer (AE51, Aethlabs, Oakland, CA) for up to 30 consecutive days. Subjects carried the monitor on a belt during waking hours, and recharged the monitor at bedside while asleep. NO_2_ were sampled by passive personal sampler (Ogawa & Co., Pompano Beach, FL) and analyzed by electron absorption spectroscopy. Then the integrated 24-h NO_2_ data were obtained for up to 30 consecutive days. Data cleaning algorithms were applied to address artifactual high and low BC readings from the aethalometer [16, 17].

### Lag periods

FeNO data and buccal cell samples were collected around 4 p.m. BC at lag 0–6 h and lag 7–12 h indicated the average BC from 10 a.m. to 4 p.m. and 4 a.m. to 10 a.m. on the same day when collecting FeNO. BC at lag 13–24 h and lag 0–24 h indicated the average BC from 4 p.m. 1 day prior to FeNO measurement to 4 a.m. and to 4 p.m. on the same day when collecting FeNO. BC at lag 25–48 h indicated the average BC from 4 p.m. 2 days to 4 p.m. 1 day prior to FeNO measurement. BC at lag 49–72 h indicated the average BC from 4 p.m. 3 days to 4 p.m. 2 day prior to FeNO measurement. BC at lag 72–96 h indicated the average BC from 4 p.m. 4 days to 4 p.m. 3 day prior to FeNO measurement.

### Covariates

Demographic information, including age, gender, and race/ethnicity, were obtained through questionnaires completed by parent or legal guardiansat the beginning of the study.

### Statistical analysis

We used SAS software version 9.4 for all analyses. Descriptive analyses were conducted to understand the distributions of air pollution, DNA methylation at each locus and FeNO measurements in general and by subject characteristics. Outcome and exposure were treated as continuous variables in all models. Spearman correlation assessed the associations of percent methylation between loci in the same gene. Since a significantly high correlation of DNA methylations between loci was observed, we applied multivariate analysis of variance (MANOVA) in generalized linear models to study the association between percent methylation and BC exposure while controlling for the correlation of %5mC among loci in the same gene (Supplement Table 1–5).

**Fig. 1:**
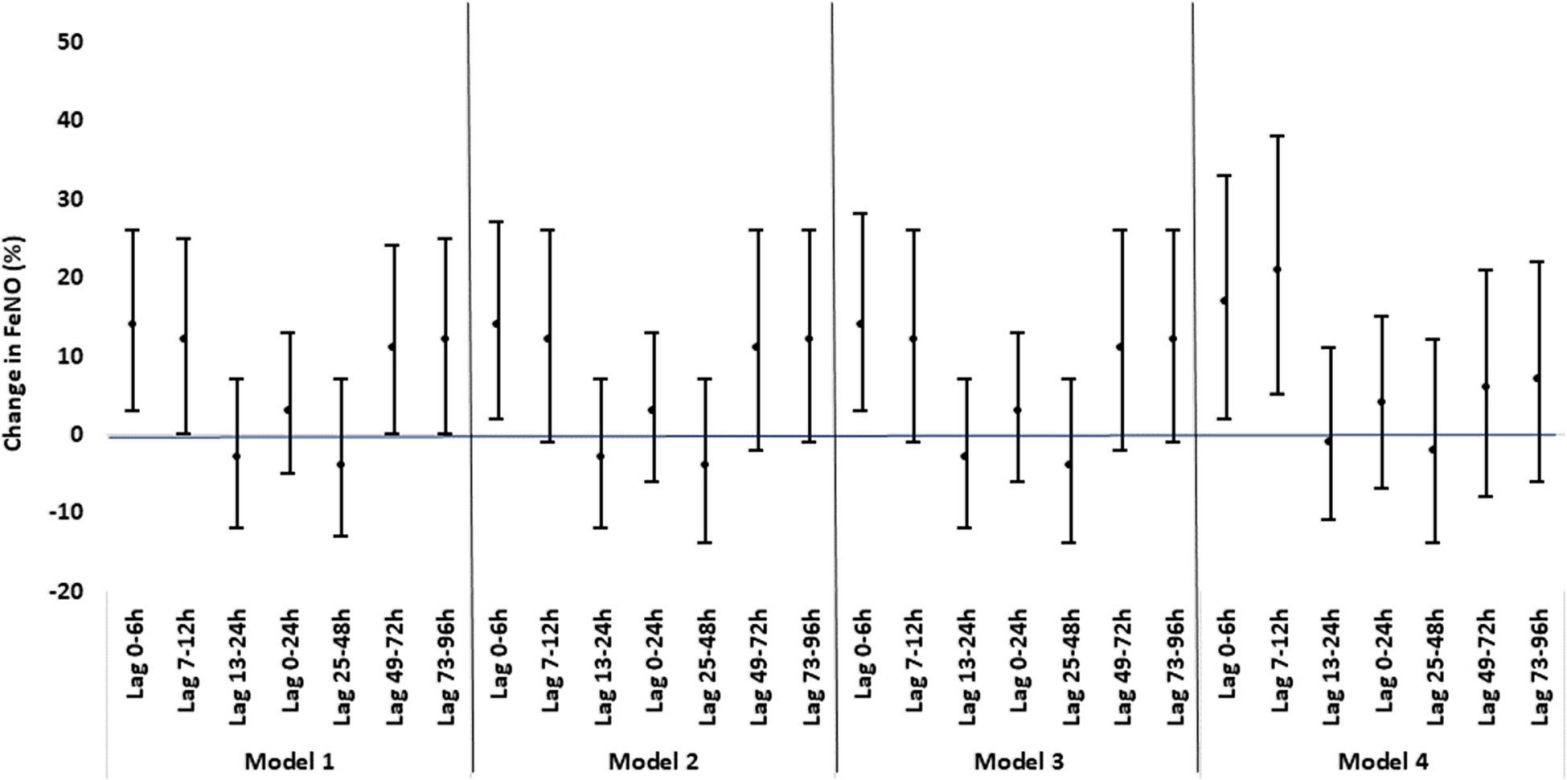
Percent Change of FeNO (and 95% CIs) per Log-transformed IQR Increase in BC at Different Lag Period. Depending on the lag period and model, the number of observations ranged from 64 to 89. Model 1 was the unadjusted model. Model 2 adjusted for day-of-week and week number when FeNO data were collected. Model 3 further adjusted for age, gender and race/ethnicity. Model 4 further adjusted for NO_2_. The IQR (μg/m^3^) was 3.83 for lag 0-6 h, 3.91 for lag 7-12 h, 3.07 for lag 13-24 h, 2.45 for lag 0-24 h, 3.42 for lag 25-48 h, 3.43 for lag 49-72 h and 2.95 for lag 73-96 h

For genes with statistically significant results in MANOVA tests, we fitted four linear mixed effect models to assess the effects of BC on FeNO and percent DNA methylation at multiple periods prior to the buccal sample collection (Fig. 1): 0–6 h, 7–12 h, 13–24 h, 0–24 h (lag 0 day), 25–48 h (lag 1 day) 49–72 h (lag 2 days) 73–96 h (lag 3 days). The four models include: unadjusted model (model 1); model adjusted for week number and day-of-week when collecting the outcomes (model 2); model further adjusted for age, gender and race/ethnicity (model 3) and model further adjusted for NO_2_ (model 4). BC and all covariates were incorporated as fixed-effect terms while a random-effect term for each subject was added to account for the correlations of repeated measurements collected from the same subject.

All tests were 2-sided at a 5% significance level. The effect estimates of DNA methylation were presented as the change and its 95% confidence interval (CIs) in %5mC per log-transformed and lag-specific interquartile range (IQR) increase in BC. The effect estimates of FeNO were presented as the relative change and its 95% CIs per log-transformed and lag-specific IQR increase in BC.

Missing data problems are usually addressed by including only subjects without missing data in any variables required for an analysis. Such method is subject to bias and loss of information [18]. We conducted a sensitivity analysis by using multiple imputation for missing BC and NO_2_ data and fitted the same models to examine the lag patterns of imputed BC, DNA methylation and FeNO levels (Supplement Table 7–8).

## Results

Table 1 summarizes the basic personal characteristics of the participating subjects. On average, children were 10 years old, and 72% (*n* = 13) were boys and 55% (*n* = 10) were Hispanic. Distribution of percent methylation of CpG loci in NOS and ARG genes is shown in Supplement Table 1. The CpG loci located in intron 1 in *NOS3* (position 1–3), 5-upstream in *NOS2A* (position 12, 13) and 5-untranslated region (5-UTR) in *NOS1* (position 1–6) and *NOS2A* (position 14, 15) were methylated > 65% while the loci located in intron 2 in *NOS2A* (position 3–11) and 5-UTR in *ARG2* (position 1–13) were nearly unmethylated (mean %5mC < 3%). The average percent methylation of CpG sites in 5-upstream in *ARG1* ranged from 5 to 91%. The between-subject correlations of methylation at each locus varied by genes. Pearson correlations ranged from − 0.08 to 0.79, − 0.59 to 0.91, 0.40 to 0.76 and − 0.75 to 0.91 of the 6 sites in *NOS1*, the 16 sites in *NOS2A*, the 3 sites in *NOS3* and 8 sites in *ARG1*, respectively (Supplement Table 2–5).

**Table 1:** Descriptive statistics of the study population (*n* = 18)

Characteristic	Measure
Age (years, mean ± SD)	10.6 ± 1.7
Male n(%)	13 (72.2)
Race/ethnicity n(%)	
African American	5 (27.8)
Hispanic	10 (55.6)
White	3 (16.6)
Maternal education n(%)	
< High school	2 (12.5)
High school graduate	9 (56.3)
Some college	5 (31.2)

The 24-h average BC prior to buccal sample collections ranged from 0.17 μg/m^3^ to 6.99 μg/m^3^, with an average of 1.63 μg/m^3^ (Table 2). The level of FeNO varied substantially from 2.5 ppb to 106 ppb with a mean of 30.6 ppb and a median of 17 ppb.

**Table 2:** Distribution of BC, daily average NO_2_, and FeNO

		N	Mean	SD	IQR	25th	Median	75th	Min	Max
BC (μg/m^3^)	without MI	90	1.6	1.4	1.1	0.8	1.1	1.9	0.2	7.0
	with MI	90	1.4	1.2	1.0	0.7	1.0	1.6	0.2	6.2
NO_2_(ppb)	without MI	74	40.6	25.1	27.9	23.3	36.7	51.2	3.4	138.3
	with MI	90	41.7	23.2	26.2	25.3	39.3	51.5	3.4	138.3
FeNO (ppb)		89	30.6	28.3	42.0	9.0	17.0	51.0	2.5	106.0

BC data were measured 24-h before buccal sample collection

*Abbreviations: BC* black carbon, *NO2* nitrogen dioxide, *FeNO* fractional exhaled nitric oxide, *MI* multiple imputation, *IQR* interquartile range

Table 3 and Fig. 1 summarize the effects of exposure to BC at various lag periods on FeNO using linear mixed-effect models. In the single-pollutant model, we found a 14% increase (95%CI: 3–26%) in FeNO per log-transformed IQR increase in BC at lag 0–6 h (*p* = 0.01). The strength of associations decreased and became insignificant at longer lag periods. This trend remains after adjusting for age, gender, race/ethnicity, week number and day-of-week for FeNO data collection. In model 4, where we further adjusted for daily NO_2_ level, we observed a 17% increase (95%CI: 3–28%) in FeNO at lag 0–6 h (*p* = 0.02) and 21% increase (95%CI: 5–38%) in FeNO at lag 7–12 h (p = 0.01), both of which are stronger compared to the strength of association from single-pollutant models. The strength decreased and became insignificant at longer lag periods.

**Table 3:** Relative changes in FeNO per log-transformed IQR increase in BC at different lag periods

Lag	Model 1		p	Model 2		p	Model 3		p	Model 4		p
	n	Relative change in FeNO (95%CI)		n	Relative change in FeNO (95%CI)		n	Relative change in FeNO (95%CI)		n	Relative change in FeNO (95%CI)	
Lag 0-6 h	89	1.14 (1.03, 1.26)	**0.01**	89	1.14 (1.02, 1.27)	**0.02**	89	1.14 (1.03, 1.28)	**0.02**	73	1.17 (1.02, 1.33)	**0.02**
Lag 7-12 h	89	1.12 (1.00, 1.25)	0.06	89	1.12 (0.99, 1.26)	0.07	89	1.12 (0.99, 1.26)	0.07	73	1.21 (1.05, 1.38)	**0.01**
Lag 13-24 h	89	0.97 (0.88, 1.07)	0.55	89	0.97 (0.88, 1.07)	0.55	89	0.97 (0.88, 1.07)	0.54	73	0.99 (0.89, 1.11)	0.91
Lag 0-24 h	89	1.03 (0.95, 1.13)	0.45	89	1.03 (0.94, 1.13)	0.52	89	1.03 (0.94, 1.13)	0.53	73	1.04 (0.93, 1.15)	0.49
Lag 25-48 h	86	0.96 (0.87, 1.07)	0.47	86	0.96 (0.86, 1.07)	0.49	86	0.96 (0.86, 1.07)	0.47	70	0.98 (0.86, 1.12)	0.75
Lag 49-72 h	83	1.11 (1.00, 1.24)	0.06	83	1.11 (0.98, 1.26)	0.09	83	1.11 (0.98, 1.26)	0.09	67	1.06 (0.92, 1.21)	0.44
Lag 73-96 h	80	1.12 (1.00, 1.25)	0.06	80	1.12 (0.99, 1.26)	0.07	80	1.12 (0.99, 1.26)	0.07	64	1.07 (0.94, 1.22)	0.32

Note: All estimates are from linear mixed effect model to reflect percent changes in FeNO per IQR increase in log-transformed BC. The IQR (μg/m^3^) was 1.8 for lag 0-6 h, 1.8 for lag 7-12 h, 1.2 for lag 13-24 h, 1.1 for lag 0-24 h, 1.5 for lag 25-48 h, 1.6 for lag 49-72 h and 1.3 for lag 73-96 h

Model 1 was the unadjusted model

Model 2 adjusted for day-of-week and week number when FeNO data were collected

Model 3 further adjusted for age, gender and race/ethnicity

Model 4 further adjusted for NO_2_

We applied the MANOVA test and found significant associations between percent methylation in the *NOS3* gene and exposure to BC at two lag periods: 13–24 h (Wilks’ Lambda *p* = 0.04) and 0–24 h (Wilks’ Lambda p = 0.04) before buccal cell sample collection (Supplement Table 6). Thus, only *NOS3* gene methylation data were used in the following analysis.

**Fig. 2:**
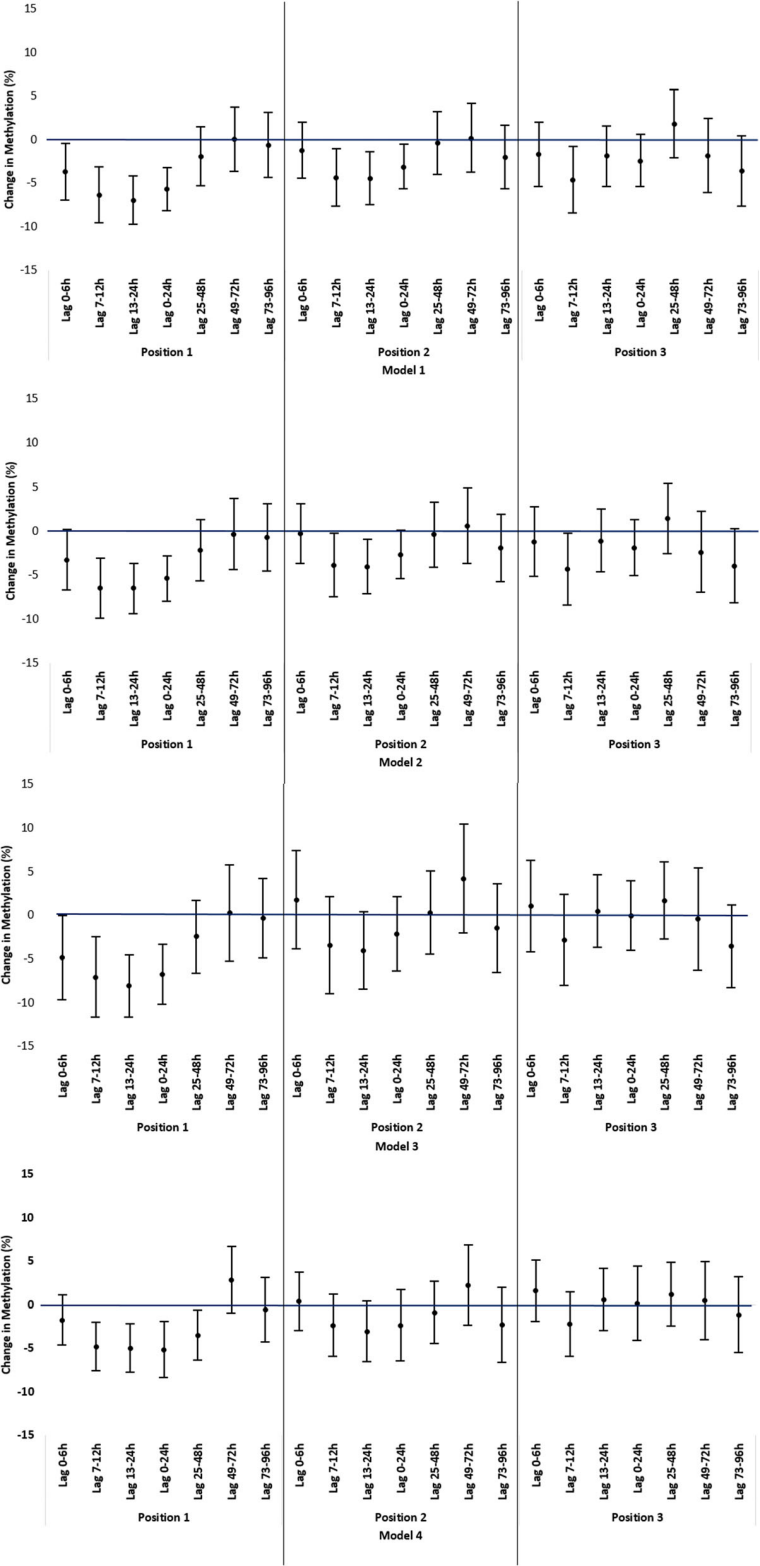
Change of Methylation in *NOS3* Gene per Log-transformed IQR Increase in BC at Different Lag Period in Model 1–4. Depending on the lag period, position of the loci and the model, the number of observations ranged from 62 to 86. Model 1 was the unadjusted model. Model 2 adjusted for day-of-week and week number when FeNO data were collected. Model 3 further adjusted for day-of-week, week number when FeNO data were collected, age, gender and race/ethnicity. Model 4 further adjusted for NO_2_

BC concentrations, in lag 7–12 h, lag 13–24 h and 0–24 h, were negatively associated with the percent methylation in Position 1 in all mixed effect models (Table 4). At lag 7–12 h, there was a 4.81% (95%CI: − 7.64, − 1.99%) to 7.13% (95%CI: − 11.76, − 2.50%) decreased methylation in Position 1 per log-transformed IQR increase in BC in model 1–4. At lag 13–24 h, a log-transformed IQR increase in BC in model 1–4 was associated with a 4.99% (95%CI: − 7.80 -2.17%) to 8.12% (95%CI: − 11.68 -4.56%) decreased methylation in Position 1. At lag 0 day, there was a 5.44% (95%CI: − 8.07, − 2.82%) to 6.79% (95%CI: − 10.27, − 3.31%) decreased methylation in Position 1 per log-transformed IQR increase in BC in all regression models. In the single-pollutant model, a log-transformed IQR increase in BC at lag 13–24 h and lag 0 day was associated with 4.47% (95%CI: − 7.51 -1.43%) and 3.13% (95%CI: − 5.73 -0.53%) decreased methylation in Position 2. The strength of association became weaker and insignificant in model 4. No significant associations were found in Position 3. BC concentrations at other lag periods were also tested to reveal the lag structure of changes in *NOS3* methylation by BC (Fig. 2). We observed a decrease in methylation level in Position 1 immediately after exposure to BC (0–6 h) in the unadjusted model 1. The methylation level became even lower at lag 7-12 h and lag 13-24 h, and then attenuated at longer lag periods. This trend remains for the adjusted models. Similar lag structures were observed for Position 2 and 3. In model 4, we found significant decrease in methylation level in Position 1 in response to BC exposure within the first 48 h after BC exposure. The associations became insignificant at longer lag periods.

**Table 4:** Percent Change of DNA Methylation in *NOS3* Gene per log-transformed IQR increase in BC at different lag periods

Lag period	***NOS3*** Gene loci	Model 1			Model 2			Model 3			Model 4		
		Difference in % methylation	95%CI	p	Difference in % methylation	95%CI	p	Difference in % methylation	95%CI	p	Difference in % methylation	95%CI	p
**Lag 0-6 h**	**Position 1**	−3.73	(−6.98, − 0.47)	**0.03**	−3.31	(−6.75, 0.13)	0.06	−4.90	(−9.71, − 0.08)	**0.05**	−1.75	(−4.66, 1.16)	0.23
	**Position 2**	−1.23	(−4.48, 2.02)	0.45	−0.36	(−3.79, 3.08)	0.84	1.75	(−3.90, 7.39)	0.54	0.37	(−3.01, 3.75)	0.83
	**Position 3**	−1.70	(−5.44, 2.04)	0.37	−1.26	(−5.24, 2.72)	0.53	1.02	(−4.26, 6.29)	0.70	1.59	(−1.94, 5.13)	0.37
	**Average**	−2.17	(−4.94, 0.61)	0.12	−1.58	(−4.52, 1.36)	0.29	−0.70	(−4.92, 3.52)	0.74	−0.11	(−2.79, 2.56)	0.93
**Lag 7-12 h**	**Position 1**	−6.38	(−9.61, − 3.15)	**< 0.01**	−6.51	(−9.93, − 3.08)	**< 0.01**	−7.13	(−11.76, − 2.50)	**< 0.01**	−4.81	(−7.64, − 1.99)	**< 0.01**
	**Position 2**	−4.38	(−7.70, − 1.06)	**0.01**	−3.92	(−7.54, − 0.29)	**0.03**	−3.49	(−9.07, 2.10)	0.21	−2.39	(−5.98, 1.19)	0.19
	**Position 3**	−4.65	(−8.49, − 0.80)	**0.02**	−4.40	(−8.54, − 0.26)	**0.04**	−2.88	(−8.09, 2.34)	0.27	−2.25	(−5.94, 1.44)	0.23
	**Average**	−5.31	(−8.03, − 2.59)	**< 0.01**	−5.51	(−8.40, − 2.61)	**< 0.01**	−4.57	(−8.61, − 0.53)	**0.03**	−3.13	(−5.84, − 0.43)	**0.02**
**Lag 13-24 h**	**Position 1**	−6.96	(−9.78, − 4.14)	**< 0.01**	−6.56	(− 9.45, − 3.68)	**< 0.01**	−8.12	(−11.68, − 4.56)	**< 0.01**	−4.99	(−7.8, − 2.17)	**< 0.01**
	**Position 2**	−4.47	(−7.51, − 1.43)	**< 0.01**	−4.09	(−7.20, − 0.98)	**0.01**	−4.05	(−8.49, 0.39)	0.07	−3.05	(−6.58, 0.47)	0.09
	**Position 3**	−1.90	(−5.41, 1.60)	0.28	−1.14	(−4.74, 2.46)	0.53	0.43	(−3.75, 4.62)	0.84	0.60	(−3.02, 4.21)	0.74
	**Average**	−4.58	(−7.02, − 2.14)	**< 0.01**	−4.37	(− 6.92, − 1.82)	**< 0.01**	−3.31	(−6.57, − 0.04)	**0.05**	−2.65	(−5.35, 0.05)	0.05
**Lag 0-24 h**	**Position 1**	−5.73	(−8.25, − 3.21)	**< 0.01**	−5.44	(−8.07, − 2.82)	**< 0.01**	−6.79	(−10.27, − 3.31)	**< 0.01**	−5.16	(−8.43, − 1.89)	**< 0.01**
	**Position 2**	−3.13	(−5.73, − 0.53)	**0.02**	−2.71	(−5.45, 0.03)	0.05	−2.17	(−6.42, 2.08)	0.31	−2.37	(−6.45, 1.70)	0.25
	**Position 3**	−2.44	(−5.46, 0.58)	0.11	−1.96	(−5.16, 1.23)	0.22	−0.08	(−4.06, 3.90)	0.97	0.15	(−4.1, 4.40)	0.94
	**Average**	−3.67	(−5.79, − 1.54)	**< 0.01**	−3.72	(−5.97, − 1.46)	**< 0.01**	−2.71	(−5.82, 0.40)	0.09	−2.69	(−5.82, 0.44)	0.09
**Lag 25-48 h**	**Position 1**	−1.94	(−5.35, 1.48)	0.26	−2.25	(−5.73, 1.23)	0.20	−2.48	(−6.66, 1.70)	0.24	−3.48	(−6.38, − 0.57)	**0.02**
	**Position 2**	−0.40	(−4.06, 3.26)	0.83	−0.44	(−4.16, 3.27)	0.81	0.29	(−4.50, 5.09)	0.90	−0.87	(−4.45, 2.71)	0.63
	**Position 3**	1.81	(−2.13, 5.76)	0.36	1.36	(−2.68, 5.40)	0.50	1.66	(−2.81, 6.12)	0.46	1.19	(−2.49, 4.87)	0.52
	**Average**	−0.50	(−3.52, 2.51)	0.74	− 0.52	(−3.57, 2.53)	0.73	−0.10	(−3.69, 3.48)	0.95	−1.37	(− 4.13, 1.40)	0.32
**Lag 49-72 h**	**Position 1**	0.02	(−3.71, 3.74)	0.99	−0.41	(−4.47, 3.64)	0.84	0.21	(−5.3, 5.72)	0.94	2.81	(−1.03, 6.66)	0.15
	**Position 2**	0.17	(−3.81, 4.14)	0.93	0.54	(−3.79, 4.86)	0.80	4.15	(−2.13, 10.43)	0.19	2.24	(−2.41, 6.89)	0.34
	**Position 3**	−1.87	(−6.16, 2.42)	0.39	−2.44	(−7.07, 2.20)	0.30	−0.46	(−6.35, 5.43)	0.87	0.47	(−4.01, 4.96)	0.83
	**Average**	−0.81	(−4.11, 2.49)	0.62	−0.76	(−4.3, 2.79)	0.67	1.32	(−3.4, 6.04)	0.58	1.95	(−1.56, 5.46)	0.27
**Lag 73-96 h**	**Position 1**	−0.63	(−4.35, 3.10)	0.74	−0.76	(−4.61, 3.10)	0.69	−0.39	(−4.98, 4.21)	0.87	−0.59	(−4.33, 3.15)	0.75
	**Position 2**	−2.04	(−5.71, 1.64)	0.27	−1.99	(−5.82, 1.84)	0.30	−1.50	(−6.62, 3.63)	0.56	−2.32	(−6.67, 2.04)	0.29
	**Position 3**	−3.62	(−7.72, 0.48)	0.08	−4.00	(− 8.25, 0.24)	0.06	−3.60	(−8.38, 1.18)	0.14	−1.14	(−5.53, 3.25)	0.60
	**Average**	−2.36	(−5.44, 0.71)	0.13	−2.45	(− 5.67, 0.77)	0.13	−1.90	(−5.78, 1.98)	0.33	−1.72	(−5.13, 1.68)	0.31

Note: All estimates are from linear mixed effect model to reflect percent changes in methylation per IQR increase in log-transformed BC. The IQR (μg/m^3^) was 1.8 for lag 0-6 h, 1.8 for lag 7-12 h, 1.2 for lag 13-24 h, 1.1 for lag 0-24 h, 1.5 for lag 25-48 h, 1.6 for lag 49-72 h and 1.3 for lag 73-96 h

Model 1 was the unadjusted model

Model 2 adjusted for day-of-week and week number when buccal cells were collected

Model 3 further adjusted for age, gender and race/ethnicity

Model 4 further adjusted for NO_2_

There was a small fraction (7–9%) of missing data for BC during the selected lag periods and 17% missing data for daily NO_2_. We used multiple imputation to fill in the missing BC and NO_2_ data. The mean (SD) of 24-h average BC and NO_2_ with vs. without imputation were 1.63 μg/m^3^ (1.36) vs. 1.43 μg/m^3^ (1.22) and 40.63 ppb (25.07) vs. 41.67 ppb (23.17), respectively (Table 2). From MANOVA test results, the same gene and lag BC periods were identified using the imputed BC (Supplement Table 6). We found similar associations between FeNO and BC with or without imputation (Supplement Table 7). Compared with the non-imputed BC, the imputed BC had slightly stronger negative associations with the methylation in NOS3 in all models (Supplement Table 8).

We also tested the associations between percent methylation in *NOS* and *ARG1* genes and FeNO at lag 1 day. In the unadjusted model, we observed a 3% (95%CI: 0–6%) increase in FeNO per percent increase in methylation in *ARG1* Position 2. We did not find statistically significant associations with methylation at other positions in *NOS* and *ARG* genes.

## Discussion

We found that personal exposure to BC, as a marker for TRAP, was positively associated with FeNO at lag periods of less than 12 h in children with asthma. To better understand the mode of action by which exposure to TRAP may increase FeNO, we examined associations between exposure to BC and DNA methylation levels in promoter regions of *NOS* and *ARG* genes. We found that exposure to BC was negatively associated only with methylation of the *NOS3* gene, suggesting that modulation of this “constitutive” isoform of NOS may be involved in short-term changes in FeNO associated with exposure to TRAP.

Consistent with previous studies, we observed a significant increase in FeNO in response to BC exposure in children with asthma. As a non-invasive biomarker of inflammation, FeNO has been reported to be positively associated with TRAP in epidemiological studies, especially in patients with preexisting asthma and COPD [3, 8] . Nitric oxide normally functions as a key physiological mediator in human immune responses and smooth muscle relaxation. However, overproduction of NO may mediate cellular toxicity and cause inflammation [19, 20]. Airway inflammation is a key mechanism in the pathogenesis of asthma exacerbation [21]. However, the biological mechanisms linking the TRAP exposure to airway inflammation and increased FeNO remain unclear.

Results from prior human studies have been inconsistent, but most studies revealed that exposure to particulate air pollutants decreased *NOS2A* promoter methylation and increased *ARG* methylation. In a cohort of 163 urban children, Jung et al. found that higher level of 24-h BC measured by personal monitors were associated with reduced methylation of *NOS2A* 5 days later [22]. The magnitude of association was stronger among the seroatopic and cockroach-sensitized children compared to non-sensitized children. In the Southern California Children’s Health Study cohort, Salam et al. reported that elevated 7-day average exposure to PM_2.5_ was associated with a statistically significant decrease in *NOS2A* methylation in 940 children [9]. Using the same cohort, Breton et al. found significantly lower *NOS2A* methylation and higher *NOS3* methylation in response to acute and chronic exposure to particulate matter [10]. Similar results were observed in panel studies of exposure to PM in China among 43 healthy adults and 30 adults with chronic obstructive pulmonary disease [8, 11] . However, one panel study revealed that occupational exposure to fine PM was positively associated with increased *NOS2A* methylation in 38 male boilermaker welders [23].

Based on these previous studies, we hypothesized, that exposure to TRAP would be negatively associated with DNA methylation in *NOS* genes, especially *NOS2A*, and positively associated with methylation in *ARG* genes in children with asthma exposed to BC. We found statistically significant negative associations between exposure to BC and *NOS3* promoter methylation only. In contrast, in Breton et al., PM_2.5_ was associated with increased methylation levels in the *NOS3* gene among children. Unlike previous studies, we did not find significant changes in the methylation level in other *NOS* and *ARG* isoforms in response to TRAP [10]. These inconsistent results could be due to several differences between our study and the previous studies, including differences in the study populations (children vs. adults, and asthma vs. other health conditions), exposure assessment approaches (personal vs. fixed-site monitoring) and the selection of CpG sites tested [8, 10, 11, 22, 23].

To further explore the role of DNA methylation in the associations of TRAP and FeNO in children with asthma, we examined the changes of *NOS* methylation and FeNO levels in response to TRAP at different time-lag periods. We found that the negative effect of BC on *NOS3* methylation at position 1 was *stronger* in the first 24 h. The largest effect of BC on FeNO was found within 12 h, and then both strengths of association decreased and became statistically insignificant. Unlike *NOS2A*, which is inducible, *NOS3* is expressed constitutively. In our previous controlled experimental studies, we found an immediate but transient increase in nitrite, a stable metabolite of NO, in the exhaled breath condensate (EBC) of healthy young adults after exposure to TRAP particles and adults with asthma after exposure to diesel engine exhaust [24, 25]. Such transient increases in EBC nitrite could be explained by rapid increases in either NO production or oxidation of NO to nitrite. However, in the current study, we did not find the expected association between methylation at the *NOS3* position 1 locus and FeNO at lag day 1.

Our study has several strengths. We followed our subjects for up to 30 consecutive days. This study design addresses the temporality between exposure and outcomes, and the repeated sampling of the same subject increased the study power. Furthermore, personal real-time BC measurement likely reduced exposure measurement error compared with data from fixed-site monitors that were used in most of the previous studies. The non-differential exposure measurement error could severely attenuate exposure-disease associations in epidemiological studies. For example, Niu et al. compared the associations of DNA methylation in *NOS2A* and *ARG2* with personal and central-site monitored ozone exposure and found much stronger associations of ozone using personal measurements [12]. To avoid multiple pairwise comparisons, we applied the MANOVA test at multiple CpG loci in *NOS* and *ARG* genes. To reduce the unknown impacts of time-varying patterns, we collected buccal cells and FeNO on various day-of-week. Unlike most studies that only focused on CpG sites in *NOS2A*, our study tested multiple CpG loci at all isoforms of *NOS* and *ARG*, both of which are involved in the regulation of NO production. Finally, we focused on children with asthma living in environmental justice communities. This specific subpopulation may be more sensitive to short-term changes in exposure to TRAP.

Nonetheless, this study has certain limitations. Our study had very limited sample size, which was justified using power calculation that assumed one outcome and exposure at one lag period. In the analysis, we controlled for multiple testing across responses by using a Multivariate Analysis of Variance, such that the overall test of all responses for an exposure was conducted at the 0.05 level before looking at individual responses. Therefore, our study may have been underpowered for looking at the responses relative to an exposure. Because the BC collected at each lag period were highly correlated we could not include multiple lags in the regression models at once and we were not able to conclusively say that one lag was more influential than another. Due to the limited sample size, we were unable to check if the time lag pattern between exposure to TRAP and DNA methylation level varies by the degree of asthma.

Besides DNA methylation, there are several types of epigenetic modifications, including histone modification and miRNA expression, which could be impacted by air pollution exposures and could affect the development and exacerbation of asthma [26–28]. However, this is beyond the scope of this paper and future studies should be done in assessing the impacts of personal TRAP exposure on histone mondification and miRNA levels in children with asthma. We used buccal cells as surrogates for cells from bronchial tissue due to the feasibility of collection in a community based study of children with asthma. Studies have found similar gene expression, as well as methylation level in buccal cells and cells from the respiratory tract in response to tobacco, supporting the potential of the oral epithelium as a surrogate tissue for respiratory epithelium [29, 30]. Other studies, noted above, have found associations between exposure to air pollution and changes in methylation in *NOS* genes from buccal cells [9–12, 22].

## Conclusions

In this study, we found that exposure to TRAP was associated with higher levels of FeNO and lower levels of DNA methylation in the promoter regions of the *NOS3* gene at various lag periods in children with asthma living in a seaport-adjacent community with high density of diesel truck traffic. Although these findings may not be generalizable to other populations, our results expand the understanding that DNA methylation of *NOS* genes could be an important biological mechanism in physiological responses to TRAP in children with asthma. To better understand the biological mechanism, future studies are needed to assess the interaction between the TRAP, epigenetic and genetic variation, airway production of NO and inflammation, and clinical outcomes.

## Supplementary Information

**Additional file 1: Supplement table 1.** Distribution of percent methylation of CpG loci in *NOS* and *ARG* genes (n=90). **Supplement table 2.** Between-subject correlation of percent methylation between different CpG sites in *NOS1* gene. **Supplement table 3.** Between-subject Correlation of percent methylation between CpG loci in *NOS2* gene. **Supplement table 4.** Between-subject Correlation of percent methylation between CpG loci in *NOS3* gene. **Supplement table 5.** Between-subject Correlation of percent methylation between CpG loci in ARG1 gene. **Supplement table 6.** MANOVA test results for *NOS* genes of raw and imputed BC at different lag periods. **Supplement table 7.** Proportion changes in FeNO per log-transformed IQR increase in imputed BC at different lag periods. **Supplement table 8.** Percent Change of DNA Methylation in *NOS3* Gene per log-transformed IQR increase in imputed BC at different lag periods.

## Data Availability

The datasets used and/or analysed during the current study are available from the corresponding author on reasonable request.

## Abbreviations

*ARG*: Arginase
BC: Black carbon
CO: Carbon monoxide
EBC: Exhaled breath condensate
FeNO: Fractional exhaled nitric oxide
HEI: Health Effect Institute
IQR: Interquartile range
MANOVA: Multivariate analysis of variance
NHLBI: The National Heart, Lung, and Blood Institute
NO: Nitric oxide
NOS: NO synthase
*NOS1*: Neuronal NOS
*NOS2A*: Inducible NOS
*NOS3*: Endothelial NOS
NOx: Oxides of nitrogen
PM: Particulate matter
PCR: Polymerase chain reaction
TRAP: Traffic-related air pollution
VOCs: Volatile organic compounds
%5mC: percentage of 5-methylcytosine
5-UTR: 5-untranslated region
95% CIs: 95% confidence interval
