# Supplementary material for: Exposure to traffic-related air pollution and changes in exhaled nitric oxide and DNA methylation in arginase and nitric oxide synthase in children with asthma
Source: Environ Health. 2021 Feb 11;20:12. doi: 10.1186/s12940-020-00678-8 (PMC7879528; doi:10.1186/s12940-020-00678-8)
Supplement: Supplementary file 1 — Additional file 1: Supplement table 1. Distribution of percent methylation of CpG loci in NOS and ARG genes (n=90). Supplement table 2. Between-subject correlation of percent methylation between different CpG sites in NOS1 gene. Supplement table 3. Between-subject Correlation of percent methylation between CpG loci in NOS2 gene. Supplement table 4. Between-subject Correlation of percent methylation between CpG loci in NOS3 gene. Supplement table 5. Between-subject Correlation of percent methylation between CpG loci in ARG1 gene. Supplement table 6. MANOVA test results for NOS genes of raw and imputed BC at different lag periods. Supplement table 7. Proportion changes in FeNO per log-transformed IQR increase in imputed BC at different lag periods. Supplement table 8. Percent Change of DNA Methylation in NOS3 Gene per log-transformed IQR increase in imputed BC at different lag periods. [file 12940_2020_678_MOESM1_ESM.docx]

| **Supplement Table 1.** Distribution of percent methylation of CpG loci in NOS and ARG genes (n=90) | | | | | | | | | | |
| --- | --- | --- | --- | --- | --- | --- | --- | --- | --- | --- |
|  |  |  |  |  |  |  |  |  |  |  |
| Gene loci | location | n | Mean | SD | IQR | 25th | Median | 75th | Min | Max |
| NOS1 |  |  |  |  |  |  |  |  |  |  |
| Position 1 | 5-UTR | 87 | 74.9 | 9.5 | 8.3 | 71.9 | 75.4 | 80.2 | 27.7 | 100.0 |
| Position 2 | 5-UTR | 87 | 90.9 | 7.5 | 5.2 | 89.1 | 91.9 | 94.4 | 37.1 | 98.1 |
| Position 3 | 5-UTR | 87 | 69.2 | 7.4 | 8.1 | 65.7 | 69.4 | 73.9 | 42.4 | 91.9 |
| Position 4 | 5-UTR | 87 | 79.8 | 3.7 | 2.4 | 79.2 | 80.4 | 81.6 | 62.4 | 85.5 |
| Position 5 | 5-UTR | 87 | 82.9 | 3.7 | 4.4 | 80.7 | 83.3 | 85.2 | 67.4 | 91.4 |
| Position 6 | 5-UTR | 87 | 75.9 | 4.4 | 3.4 | 74.6 | 76.6 | 78.0 | 47.3 | 85.3 |
| Average |  | 87 | 78.9 | 4.4 | 4.1 | 77.1 | 80.0 | 81.2 | 59.4 | 90.9 |
| NOS2 |  |  |  |  |  |  |  |  |  |  |
| Position 1 | Intron 1 | 89 | 53.8 | 9.0 | 5.5 | 50.9 | 54.0 | 56.3 | 0.0 | 98.6 |
| Position 2 | Intron 1 | 89 | 79.4 | 6.5 | 6.4 | 76.1 | 79.7 | 82.5 | 55.3 | 99.0 |
| Position 3 | Intron 2 | 90 | 1.0 | 0.7 | 1.2 | 0.5 | 1.0 | 1.6 | 0.0 | 3.0 |
| Position 4 | Intron 2 | 90 | 0.6 | 0.5 | 0.9 | 0.0 | 0.6 | 0.9 | 0.0 | 2.3 |
| Position 5 | Intron 2 | 90 | 2.8 | 1.7 | 1.4 | 2.1 | 2.8 | 3.5 | 0.0 | 10.7 |
| Position 6 | Intron 2 | 90 | 1.8 | 1.4 | 1.5 | 1.0 | 1.9 | 2.5 | 0.0 | 8.7 |
| Position 7 | Intron 2 | 90 | 1.3 | 0.9 | 1.9 | 0.0 | 1.3 | 1.9 | 0.0 | 3.7 |
| Position 8 | Intron 2 | 90 | 1.6 | 1.0 | 1.2 | 1.1 | 1.6 | 2.3 | 0.0 | 5.7 |
| Position 9 | Intron 2 | 90 | 1.3 | 0.8 | 0.9 | 0.9 | 1.4 | 1.8 | 0.0 | 3.9 |
| Position 10 | Intron 2 | 90 | 0.9 | 0.7 | 1.4 | 0.0 | 1.1 | 1.4 | 0.0 | 2.6 |
| Position 11 | Intron 2 | 90 | 1.1 | 0.8 | 1.6 | 0.0 | 1.3 | 1.6 | 0.0 | 3.7 |
| Position 12 | 5-Upstream | 87 | 91.2 | 3.5 | 2.7 | 89.8 | 91.1 | 92.5 | 73.9 | 100.0 |
| Position 13 | 5-Upstream | 87 | 95.6 | 3.3 | 5.0 | 93.2 | 96.4 | 98.2 | 86.7 | 100.0 |
| Position 14 | 5-UTR | 87 | 82.6 | 8.0 | 8.4 | 79.2 | 83.4 | 87.6 | 42.2 | 100.0 |
| Position 16 | 5-UTR | 87 | 88.6 | 11.0 | 3.5 | 88.6 | 90.6 | 92.2 | 0.0 | 96.9 |
| Average |  | 90 | 32.8 | 4.7 | 1.1 | 33.1 | 33.8 | 34.2 | 0.0 | 35.8 |
| NOS3 |  |  |  |  |  |  |  |  |  |  |
| Position 1 | Intron 1 | 86 | 72.3 | 10.9 | 9.1 | 67.9 | 72.0 | 77.0 | 16.6 | 100.0 |
| Position 2 | Intron 1 | 86 | 76.5 | 11.1 | 8.9 | 72.2 | 78.8 | 81.1 | 16.7 | 100.0 |
| Position 3 | Intron 1 | 86 | 66.3 | 12.3 | 17.0 | 57.5 | 70.4 | 74.5 | 32.7 | 87.1 |
| Average |  | 86 | 71.7 | 9.2 | 10.9 | 65.9 | 74.2 | 76.8 | 39.2 | 91.9 |
| ARG1 |  |  |  |  |  |  |  |  |  |  |
| Position 1 | 5-Upstream | 90 | 86.9 | 5.1 | 6.4 | 83.8 | 88.0 | 90.2 | 72.8 | 96.7 |
| Position 2 | 5-Upstream | 90 | 91.3 | 2.8 | 3.4 | 89.9 | 91.6 | 93.2 | 84.2 | 98.6 |
| Position 3 | 5-Upstream | 90 | 9.3 | 3.3 | 3.6 | 7.9 | 9.1 | 11.4 | 0.0 | 16.7 |
| Position 4 | 5-Upstream | 90 | 24.6 | 6.1 | 8.7 | 20.6 | 25.1 | 29.2 | 3.1 | 36.0 |
| Position 5 | 5-Upstream | 90 | 5.6 | 2.2 | 2.5 | 4.5 | 5.6 | 7.0 | 0.0 | 10.2 |
| Position 6 | 5-Upstream | 90 | 44.4 | 16.9 | 23.4 | 33.3 | 42.5 | 56.7 | 0.0 | 100.0 |
| Position 7 | Exon 1 | 90 | 71.3 | 10.0 | 8.0 | 67.5 | 72.4 | 75.5 | 5.2 | 100.0 |
| Position 8 | Intron 1 | 90 | 54.3 | 7.8 | 10.7 | 49.2 | 54.8 | 59.9 | 33.1 | 82.0 |
| Average |  | 90 | 48.5 | 2.6 | 2.9 | 47.1 | 48.4 | 50.0 | 36.6 | 54.7 |
| ARG2 |  |  |  |  |  |  |  |  |  |  |
| Position 1 | 5-UTR | 87 | 0.6 | 1.3 | 0.0 | 0.0 | 0.0 | 0.0 | 0.0 | 5.4 |
| Position 2 | 5-UTR | 87 | 0.8 | 1.3 | 1.9 | 0.0 | 0.0 | 1.9 | 0.0 | 4.7 |
| Position 3 | 5-UTR | 87 | 0.2 | 1.3 | 0.0 | 0.0 | 0.0 | 0.0 | 0.0 | 11.8 |
| Position 4 | 5-UTR | 87 | 0.1 | 0.4 | 0.0 | 0.0 | 0.0 | 0.0 | 0.0 | 2.5 |
| Position 5 | 5-UTR | 87 | 0.0 | 0.2 | 0.0 | 0.0 | 0.0 | 0.0 | 0.0 | 1.6 |
| Position 6 | 5-UTR | 87 | 0.2 | 0.7 | 0.0 | 0.0 | 0.0 | 0.0 | 0.0 | 3.0 |
| Position 7 | 5-UTR | 87 | 0.0 | 0.0 | 0.0 | 0.0 | 0.0 | 0.0 | 0.0 | 0.0 |
| Position 8 | 5-UTR | 87 | 0.1 | 0.7 | 0.0 | 0.0 | 0.0 | 0.0 | 0.0 | 6.0 |
| Position 9 | 5-UTR | 87 | 0.0 | 0.0 | 0.0 | 0.0 | 0.0 | 0.0 | 0.0 | 0.0 |
| Position 10 | 5-UTR | 87 | 0.0 | 0.0 | 0.0 | 0.0 | 0.0 | 0.0 | 0.0 | 0.0 |
| Position 11 | 5-UTR | 87 | 0.0 | 0.0 | 0.0 | 0.0 | 0.0 | 0.0 | 0.0 | 0.0 |
| Position 12 | 5-UTR | 87 | 0.3 | 1.8 | 0.0 | 0.0 | 0.0 | 0.0 | 0.0 | 16.3 |
| Position 13 | 5-UTR | 87 | 0.0 | 0.0 | 0.0 | 0.0 | 0.0 | 0.0 | 0.0 | 0.0 |
| Average |  | 87 | 0.2 | 0.2 | 0.3 | 0.0 | 0.0 | 0.3 | 0.0 | 1.3 |
| Abbreviations: IQR, interquartile range; Max, maximum; Min minimum; 5-UTR, 5-untranslated region. 25th and 75th are percentiles | | | | | | | | | | |
|  |  |  |  |  |  |  |  |  |  |  |

| **Supplement Table 2.** Between-subject correlation of percent methylation between different CpG sites in NOS1 gene | | | | | | |
| --- | --- | --- | --- | --- | --- | --- |
|  |  |  |  |  |  |  |
|  | Position 1 | Position 2 | Position 3 | Position 4 | Position 5 | Position 6 |
| Position 1 | 1.00 |  |  |  |  |  |
| Position 2 | 0.60 | 1.00 |  |  |  |  |
| Position 3 | 0.70 | 0.36 | 1.00 |  |  |  |
| Position 4 | 0.41 | 0.54 | -0.08 | 1.00 |  |  |
| Position 5 | 0.72 | 0.32 | 0.34 | 0.33 | 1.00 |  |
| Position 6 | 0.71 | 0.35 | 0.15 | 0.59 | 0.79 | 1.00 |
|  |  |  |  |  |  |  |

| **Supplement Table 3.** Between-subject Correlation of percent methylation between CpG loci in NOS2 gene | | | | | | | | | | | | | | | | |
| --- | --- | --- | --- | --- | --- | --- | --- | --- | --- | --- | --- | --- | --- | --- | --- | --- |
|  |  |  |  |  |  |  |  |  |  |  |  |  |  |  |  |  |
|  | Position 1 | Position 2 | Position 3 | Position 4 | Position 5 | Position 6 | Position 7 | Position 8 | Position 9 | Position 10 | Position 11 | Position 12 | Position 13 | Position 14 | Position 15 | Position 16 |
| Position 1 | 1.00 |  |  |  |  |  |  |  |  |  |  |  |  |  |  |  |
| Position 2 | 0.76 | 1.00 |  |  |  |  |  |  |  |  |  |  |  |  |  |  |
| Position 3 | -0.07 | -0.29 | 1.00 |  |  |  |  |  |  |  |  |  |  |  |  |  |
| Position 4 | -0.14 | -0.44 | 0.80 | 1.00 |  |  |  |  |  |  |  |  |  |  |  |  |
| Position 5 | 0.26 | -0.02 | 0.77 | 0.69 | 1.00 |  |  |  |  |  |  |  |  |  |  |  |
| Position 6 | -0.03 | -0.29 | 0.90 | 0.75 | 0.79 | 1.00 |  |  |  |  |  |  |  |  |  |  |
| Position 7 | -0.05 | -0.20 | 0.82 | 0.82 | 0.74 | 0.76 | 1.00 |  |  |  |  |  |  |  |  |  |
| Position 8 | -0.30 | -0.35 | 0.82 | 0.83 | 0.59 | 0.72 | 0.85 | 1.00 |  |  |  |  |  |  |  |  |
| Position 9 | -0.31 | -0.28 | 0.79 | 0.74 | 0.58 | 0.72 | 0.84 | 0.90 | 1.00 |  |  |  |  |  |  |  |
| Position 10 | -0.16 | -0.12 | 0.75 | 0.69 | 0.55 | 0.71 | 0.87 | 0.84 | 0.91 | 1.00 |  |  |  |  |  |  |
| Position 11 | 0.09 | 0.08 | 0.63 | 0.64 | 0.53 | 0.63 | 0.81 | 0.72 | 0.68 | 0.83 | 1.00 |  |  |  |  |  |
| Position 12 | -0.43 | -0.39 | -0.24 | -0.19 | -0.19 | -0.23 | -0.22 | -0.23 | -0.16 | -0.15 | -0.35 | 1.00 |  |  |  |  |
| Position 13 | 0.75 | 0.50 | -0.29 | -0.47 | -0.03 | -0.13 | -0.37 | -0.59 | -0.52 | -0.39 | -0.22 | -0.12 | 1.00 |  |  |  |
| Position 14 | -0.11 | -0.14 | 0.27 | 0.49 | 0.35 | 0.28 | 0.56 | 0.42 | 0.56 | 0.65 | 0.34 | 0.23 | -0.33 | 1.00 |  |  |
| Position 15 | -0.01 | -0.03 | -0.20 | 0.03 | -0.13 | -0.23 | 0.05 | -0.09 | 0.07 | 0.08 | 0.02 | 0.43 | 0.05 | 0.43 | 1.00 |  |
| Position 16 | -0.27 | -0.06 | 0.36 | 0.16 | 0.22 | 0.35 | 0.37 | 0.43 | 0.47 | 0.40 | 0.25 | -0.35 | -0.41 | 0.05 | -0.54 | 1.00 |

| **Supplement Table 4.** Between-subject Correlation of percent methylation between CpG loci in NOS3 gene | | | |
| --- | --- | --- | --- |
|  | | | |
|  | Position 1 | Position 2 | Position 3 |
| Position 1 | 1.00 |  |  |
| Position 2 | 0.57 | 1.00 |  |
| Position 3 | 0.40 | 0.76 | 1.00 |

| **Supplement Table 5.** Between-subject Correlation of percent methylation between CpG loci in ARG1 gene | | | | | | | | |
| --- | --- | --- | --- | --- | --- | --- | --- | --- |
|  | | | | | | | | |
|  | Position 1 | Position 2 | Position 3 | Position 4 | Position 5 | Position 6 | Position 7 | Position 8 |
| Position 1 | 1.00 |  |  |  |  |  |  |  |
| Position 2 | 0.62 | 1.00 |  |  |  |  |  |  |
| Position 3 | -0.10 | -0.13 | 1.00 |  |  |  |  |  |
| Position 4 | 0.31 | 0.16 | 0.73 | 1.00 |  |  |  |  |
| Position 5 | -0.09 | -0.08 | 0.91 | 0.60 | 1.00 |  |  |  |
| Position 6 | -0.75 | -0.54 | 0.02 | -0.45 | 0.16 | 1.00 |  |  |
| Position 7 | -0.49 | -0.24 | 0.40 | 0.36 | 0.39 | 0.37 | 1.00 |  |
| Position 8 | 0.59 | 0.30 | -0.20 | 0.23 | -0.24 | -0.58 | -0.08 | 1.00 |

| **Supplement Table 6.** MANOVA test results for NOS genes of raw and imputed BC at different lag periods | | | |
| --- | --- | --- | --- |
|  |  |  |  |
| **Gene** | **Lag time** | **P value from MANOVA (Wilks' Lambda) for raw BC** | **P value from MANOVA (Wilks' Lambda) for imputed BC** |
| NOS1 | 0-6h | 0.20 | 0.40 |
|  | 7-12h | 0.64 | 0.58 |
|  | 13-24h | 0.34 | 0.15 |
|  | 0-24h(lag 0d) | 0.33 | 0.30 |
|  | 25-48h(lag 1d) | 0.82 | 0.92 |
|  | 48-72h(lag 2d) | 0.29 | 0.38 |
|  | 72-96h(lag 3d) | 0.28 | 0.55 |
| NOS2 | 0-6h | 0.90 | 0.80 |
|  | 7-12h | 0.95 | 0.95 |
|  | 13-24h | 0.83 | 0.87 |
|  | 0-24h(lag 0d) | 0.90 | 0.91 |
|  | 25-48h(lag 1d) | 0.12 | 0.33 |
|  | 48-72h(lag 2d) | 0.53 | 0.76 |
|  | 72-96h(lag 3d) | 0.13 | 0.34 |
| NOS3 | 0-6h | 0.06 | 0.13 |
|  | 7-12h | 0.10 | 0.11 |
|  | 13-24h | 0.04 | 0.02 |
|  | 0-24h(lag 0d) | 0.04 | 0.04 |
|  | 25-48h(lag 1d) | 0.28 | 0.98 |
|  | 48-72h(lag 2d) | 0.29 | 0.71 |
|  | 72-96h(lag 3d) | 0.56 | 0.90 |
| ARG1 | 0-6h | 0.08 | 0.11 |
|  | 7-12h | 0.83 | 0.83 |
|  | 13-24h | 0.88 | 0.78 |
|  | 0-24h(lag 0d) | 0.51 | 0.68 |
|  | 25-48h(lag 1d) | 0.83 | 0.81 |
|  | 48-72h(lag 2d) | 0.21 | 0.30 |
|  | 72-96h(lag 3d) | 0.64 | 0.55 |

| **Supplement Table 7.** Proportion changes in FeNO per log-transformed IQR increase in imputed BC at different lag periods | | | | | | | | |
| --- | --- | --- | --- | --- | --- | --- | --- | --- |
|  |  |  |  |  |  |  |  |  |
|  | Model 1 |  | Model 2 |  | Model 3 |  | Model 4 |  |
| Lag | Relative change FeNO (95%CI) | P | Relative change FeNO (95%CI) | P | Relative change FeNO (95%CI) | P | Relative change FeNO (95%CI) | P |
| Lag 0-6h | 1.11 (0.99 , 1.26) | 0.08 | 1.12 (1.00 , 1.26) | **0.05** | 1.12 (1.00 , 1.26) | **0.05** | 1.13 (1.00 , 1.27) | **0.04** |
| Lag 7-12h | 1.10 (0.95 , 1.28) | 0.21 | 1.12 (0.99 , 1.27) | 0.08 | 1.12 (0.99 , 1.27) | 0.08 | 1.11 (0.98 , 1.27) | 0.10 |
| Lag 13-24h | 0.99 (0.89 , 1.11) | 0.88 | 0.99 (0.89 , 1.1) | 0.86 | 0.99 (0.89 , 1.1) | 0.86 | 0.99 (0.89 , 1.1) | 0.88 |
| Lag 0-24h | 1.04 (0.93 , 1.17) | 0.45 | 1.05 (0.95 , 1.16) | 0.36 | 1.05 (0.95 , 1.16) | 0.36 | 1.05 (0.95 , 1.16) | 0.37 |
| Lag 25-48h | 0.99 (0.87 , 1.14) | 0.92 | 0.98 (0.88 , 1.1) | 0.78 | 0.98 (0.88 , 1.1) | 0.76 | 0.98 (0.88 , 1.1) | 0.79 |
| Lag 49-72h | 1.11 (0.97 , 1.27) | 0.12 | 1.1 (0.98 , 1.24) | 0.11 | 1.1 (0.98 , 1.24) | 0.12 | 1.1 (0.97 , 1.24) | 0.12 |
| Lag 73-96h | 1.06 (0.95 , 1.18) | 0.28 | 1.06 (0.96 , 1.17) | 0.25 | 1.06 (0.96 , 1.17) | 0.27 | 1.05 (0.95 , 1.16) | 0.33 |

| **Supplement Table 8.** Percent Change of DNA Methylation in NOS3 Gene per log-transformed IQR increase in imputed BC at different lag periods | | | | | | | | | |
| --- | --- | --- | --- | --- | --- | --- | --- | --- | --- |
|  |  |  |  |  |  |  |  |  |  |
|  |  | Model 1 |  | Model 2 |  | Model 3 |  | Model 4 |  |
| Lag period | NOS3 Gene loci | Difference in % methylation (95%CI) | P | Difference in % methylation (95%CI) | P | Difference in % methylation (95%CI) | P | Difference in % methylation (95%CI) | P |
| Lag 0-6h | Position 1 | -4.53 (-7.88 , -1.19) | **0.01** | -4.21 (-7.67 , -0.76) | **0.02** | -3.65 (-7.15 , -0.15) | **0.04** | -3.65 (-7.17 , -0.13) | **0.04** |
|  | Position 2 | -1.75 (-5.11 , 1.61) | 0.31 | -1.05 (-4.54 , 2.44) | 0.55 | -0.12 (-3.89 , 3.66) | 0.95 | -0.14 (-3.93 , 3.65) | 0.94 |
|  | Position 3 | -2.18 (-6.01 , 1.65) | 0.27 | -1.74 (-5.73 , 2.25) | 0.39 | -1.11 (-5.31 , 3.08) | 0.60 | -1.09 (-5.3 , 3.13) | 0.61 |
|  | Average | -2.81 (-5.64 , 0.02) | 0.05 | -2.4 (-5.35 , 0.55) | 0.11 | -1.72 (-4.79 , 1.36) | 0.27 | -1.7 (-4.78 , 1.37) | 0.28 |
| Lag 7-12h | Position 1 | -6.66 (-10.08 , -3.24) | **<0.001** | -6.64 (-10.27 , -3.01) | **0.00** | -5.93 (-9.68 , -2.17) | **0.00** | -6.31 (-10.08 , -2.54) | **0.00** |
|  | Position 2 | -4.72 (-8.25 , -1.18) | **0.01** | -4.13 (-7.99 , -0.27) | **0.04** | -3.41 (-7.7 , 0.89) | 0.12 | -3.57 (-7.9 , 0.76) | 0.11 |
|  | Position 3 | -4.77 (-8.83 , -0.71) | **0.02** | -4.46 (-8.83 , -0.1) | 0.05 | -3.57 (-8.22 , 1.07) | 0.13 | -3.75 (-8.44 , 0.94) | 0.12 |
|  | Average | -5.59 (-8.48 , -2.71) | **<0.001** | -5.76 (-8.85 , -2.66) | **<0.001** | -4.62 (-7.96 , -1.27) | **0.01** | -4.82 (-8.17 , -1.47) | **0.01** |
| Lag 13-24h | Position 1 | -7.74 (-10.61 , -4.88) | **<0.001** | -7.17 (-10.17 , -4.16) | **<0.001** | -6.56 (-9.7 , -3.42) | **<0.001** | -6.56 (-9.7 , -3.42) | **<0.001** |
|  | Position 2 | -4.73 (-7.9 , -1.55) | **0.00** | -4.17 (-7.49 , -0.85) | **0.01** | -3.69 (-7.4 , 0.01) | **0.05** | -3.75 (-7.48 , -0.03) | **0.05** |
|  | Position 3 | -1.75 (-5.46 , 1.95) | 0.35 | -0.81 (-4.69 , 3.06) | 0.68 | 0.46 (-3.63 , 4.55) | 0.82 | 0.4 (-3.72 , 4.51) | 0.85 |
|  | Average | -5.05 (-7.67 , -2.43) | **<0.001** | -4.59 (-7.3 , -1.87) | **0.00** | -3.77 (-6.71 , -0.82) | **0.01** | -3.71 (-6.67 , -0.76) | **0.01** |
| Lag 0-24h | Position 1 | -6.36 (-9.05 , -3.68) | **<0.001** | -6.08 (-8.89 , -3.27) | **<0.001** | -5.49 (-8.44 , -2.55) | **<0.001** | -5.62 (-8.56 , -2.67) | **<0.001** |
|  | Position 2 | -3.61 (-6.39 , -0.82) | **0.01** | -3.11 (-6.07 , -0.16) | **0.04** | -2.53 (-5.87 , 0.81) | 0.14 | -2.61 (-5.97 , 0.76) | 0.13 |
|  | Position 3 | -2.75 (-5.99 , 0.49) | 0.10 | -2.21 (-5.66 , 1.23) | 0.21 | -1.2 (-4.92 , 2.51) | 0.53 | -1.28 (-5.02 , 2.46) | 0.50 |
|  | Average | -4.58 (-6.9 , -2.25) | **<0.001** | -4.29 (-6.72 , -1.87) | **0.00** | -3.56 (-6.22 , -0.89) | **0.01** | -3.53 (-6.19 , -0.87) | **0.01** |
| Lag 25-48h | Position 1 | -1.41 (-4.65 , 1.82) | 0.39 | -1.75 (-5.07 , 1.58) | 0.30 | -1.28 (-4.59 , 2.03) | 0.45 | -1.27 (-4.59 , 2.06) | 0.46 |
|  | Position 2 | -1.04 (-4.64 , 2.56) | 0.57 | -0.97 (-4.64 , 2.7) | 0.60 | -0.41 (-4.23 , 3.41) | 0.84 | -0.35 (-4.19 , 3.49) | 0.86 |
|  | Position 3 | 0.66 (-3.33 , 4.65) | 0.75 | 0.32 (-3.73 , 4.38) | 0.88 | 0.77 (-3.38 , 4.92) | 0.72 | 0.8 (-3.37 , 4.96) | 0.71 |
|  | Average | -0.82 (-3.8 , 2.17) | 0.59 | -0.87 (-3.87 , 2.13) | 0.57 | -0.37 (-3.42 , 2.68) | 0.81 | -0.38 (-3.43 , 2.68) | 0.81 |
| Lag 49-72h | Position 1 | 0.33 (-3.15 , 3.81) | 0.85 | 0.09 (-3.75 , 3.93) | 0.96 | 0.74 (-3.13 , 4.61) | 0.71 | 0.73 (-3.18 , 4.64) | 0.71 |
|  | Position 2 | 0.7 (-3.09 , 4.48) | 0.72 | 0.96 (-3.18 , 5.1) | 0.65 | 2.53 (-1.96 , 7.01) | 0.27 | 2.51 (-2 , 7.02) | 0.28 |
|  | Position 3 | -1.59 (-5.87 , 2.69) | 0.47 | -2.2 (-6.91 , 2.5) | 0.36 | -1.34 (-6.26 , 3.57) | 0.59 | -1.35 (-6.3 , 3.59) | 0.59 |
|  | Average | -0.46 (-3.62 , 2.71) | 0.78 | -0.5 (-3.92 , 2.92) | 0.77 | 0.48 (-3.05 , 4.01) | 0.79 | 0.45 (-3.11 , 4) | 0.80 |
| Lag 73-96h | Position 1 | 0.28 (-2.68 , 3.23) | 0.85 | -0.07 (-3.05 , 2.91) | 0.96 | 0.08 (-2.91 , 3.06) | 0.96 | -0.01 (-3.02 , 3.01) | 1.00 |
|  | Position 2 | -0.88 (-3.87 , 2.1) | 0.56 | -1.07 (-4.13 , 1.99) | 0.49 | -0.7 (-3.91 , 2.51) | 0.67 | -0.79 (-4.05 , 2.48) | 0.64 |
|  | Position 3 | -1.46 (-4.9 , 1.97) | 0.40 | -1.81 (-5.33 , 1.71) | 0.31 | -1.61 (-5.19 , 1.97) | 0.38 | -1.61 (-5.24 , 2.02) | 0.39 |
|  | Average | -0.79 (-3.4 , 1.82) | 0.55 | -1.04 (-3.66 , 1.58) | 0.44 | -0.83 (-3.47 , 1.8) | 0.54 | -0.89 (-3.56 , 1.77) | 0.51 |
